# PDGF beta targeting in cervical cancer cells suggest a fine-tuning of compensatory signalling pathways to sustain tumourigenic stimulation

**DOI:** 10.1111/jcmm.12449

**Published:** 2014-10-14

**Authors:** Oana Mihaela Tudoran, Olga Soritau, Loredana Balacescu, Laura Pop, Guillaume Meurice, Simona Visan, Staffan Lindberg, Alexandru Eniu, Ulo Langel, Ovidiu Balacescu, Ioana Berindan-Neagoe

**Affiliations:** a Department of Functional Genomics and Experimental Pathology, The Oncology Institute “Prof. Dr. I. Chiricuta”Cluj-Napoca, Romania; b Research Center for Functional Genomics, Biomedicine and Translational Medicine, University of Medicine and PharmacyCluj-Napoca, Romania; c Bioinformatic Core Facility, Institut Gustave RoussyVillejuif, Paris, France; d Department of Pathologic Anatomy, Necropsy and Veterinary Forensic Medicine, University of Agricultural Sciences and Veterinary MedicineCluj-Napoca, Romania; e Department of Neurochemistry, Stockholm UniversityStockholm, Sweden

**Keywords:** PDGFBB, cervical cancer, microarray, molecular signalling

## Abstract

The platelet-derived growth factor (PDGF) signalling pathway has been reported to play an important role in human cancers by modulating autocrine and paracrine processes such as tumour growth, metastasis and angiogenesis. Several clinical trials document the benefits of targeting this pathway; however, in cervical cancer the role of PDGF signalling in still unclear. In this study, we used siRNA against PDGF beta (PDGFBB) to investigate the cellular and molecular mechanisms of PDGFBB signalling in Ca Ski and HeLa cervical cancer cells. Our results show that PDGFBB inhibition in Ca Ski cells led to rapid alterations of the transcriptional pattern of 579 genes, genes that are known to have antagonistic roles in regulating tumour progression. Concomitantly, with the lack of significant effects on cervical cancer cells proliferation, apoptosis, migration or invasion, these findings suggests that cervical cancer cells shift between compensatory signalling pathways to maintain their behaviour. The observed autocrine effects were limited to cervical cancer cells ability to adhere to an endothelial cell (EC) monolayer. However, by inhibiting PDGFBB on cervical cells, we achieved reduced proliferation of ECs in co-culture settings and cellular aggregation in conditioned media. Because of lack of PDGF receptor expression on ECs, we believe that these effects are a result of indirect PDGFBB paracrine signalling mechanisms. Our results shed some light into the understanding of PDGFBB signalling mechanism in cervical cancer cells, which could be further exploited for the development of synergistic anti-tumour and anti-angiogenic therapeutic strategies.

## Background

Cervical cancer is the second cause of mortality in women, and one of the most aggressive gynaecological diseases 1, this aggressiveness being mainly caused by the favourable environment for rapidly inducing and sustaining prolific angiogenesis 2. To respond to increased metabolic needs, tumour cells release a complex of pro-angiogenic factors that activate signalling networks to generate new blood vessels. Expression of pro-angiogenic factors such as VEGF, platelet-derived growth factor (PDGF), fibroblast growth factor and angiopoietins is often up-regulated in malignant tissues, making them an attractive anti-angiogenic targeting strategy 3.

PDGF signalling pathway has been shown to participate in angiogenesis stimulation and recruitment of tumour stroma primarily by targeting perivascular cells such as pericytes (PCs) and vascular smooth muscle cells (VSMCs). Also, processes such as autocrine stimulation of tumour cell growth have been showed to be activated by PDGF/PDGF receptors (PDGFRs) signalling 4. PDGF family consists of four structurally related peptides that link through disulfide bonds to form homo and heterodimers, among which, the PDGF beta (PDGFBB) is described to be the most pluripotent form as it interacts to all PDGFRs. Several studies showed that PDGFBB targeting have anti-angiogenic and anti-tumour effects 5,6. The efficiency of several anti-angiogenic drugs in cervical cancer are currently under investigation in various clinical trials, either as single or multi-targeting strategy, alone or in combination with classic chemotherapeutics 7. While bevacizumab treatments seemed to be well tolerated and active in the treatment of recurrent cervical cancer 8, dual targeting VEGF and PDGF pathways with sunitinib showed no objective response as single agent 9. However, when treated with pazopanib, a small molecule drug that targets VEGF, PDGF and c-Kit pathways, an improved progression-free survival was achieved 7. These findings suggest that when PDGF signalling is turned off, alternative growth pathways may become activated as a resistance mechanism 10; therefore, further in-depth mechanistic studies are required to understand how PDGFBB regulates the tumour signalling cascades in the development and progression of cancer. To our knowledge, the role of PDGFBB molecule in cervical cancer has not yet been documented.

Using small interfering RNAs (siRNAs), a classical investigative method for functional genomics studies, we explored the molecular mechanisms and functions of PDGFBB signalling in the two main types of cervical cancer: squamous cells carcinoma represented by Ca Ski cell line and adenocarcinoma represented by HeLa cell line. In this study, we provide evidence that PDGFBB signalling inhibition leads to transcriptional regulation of hundreds of genes known to modulate cell proliferation, apoptosis, motility and adhesion. Our results suggest that autocrine PDGFBB signalling in cervical cancer is regulated by a feedback loop that allows cells to selectively activate/repress signalling pathways to maintain cervical tumour cell proliferation, survival and motility. Furthermore, by inhibiting PDGFBB expression in cervical cancer cells, we induced paracrine effects on endothelial cells (ECs) in co-culture and conditioned media systems, effects that seem to be mediated indirectly, through a shift in expression of several extracellular molecules.

## Materials and methods

### Cell culture

Human umbilical vascular endothelial cells (HUVEC) and HPV positive HeLa and Ca Ski cells were purchased from European Cell Colure Collection. HeLa cells were cultured in DMEM growth medium with 1000 mg/l glucose and Ca Ski and HUVEC cells were cultured in RPMI-1640 medium at 37°C in 5% carbon dioxide humidified air. Each culture medium was supplemented with 10% foetal bovine serum, 2 mM L-glutamine, 100 U/ml penicillin and 100 U/ml streptomycin. Additionally, 1% NEAA was added to HeLa cell culture media. All cell culture reagents were purchased from Sigma-Aldrich (St. Louis, MO, USA).

### siRNA treatment

Confluent cells (2.5 × 10^5^ ) were seeded in 12 well plates at a cell density of 10^5^ cells/ml 24 hrs before treatment. Thirty minutes before treatment, fresh siRNA nanoparticles were prepared by incubating PDGFBB siRNA (sequence 11,674, sense 5′-GGAGCUUUAUGAGAUGCUGTT-3′ antisense 5′-CAGCAUCUCAUAAAGCUCCTC-3′, Ambion by Life Technologies, Carlsbad, CA, USA) or negative control (random) siRNA (AM 4612, Ambion by Life Technologies) at 1:30 molar ratio with PepFect 6 (PF6) as previously described 11. Serum media were replaced with 900 μl of Opti-Mem (Gibco by Life Technologies) and 100 μl nanocomplexes were added to each well to a final concentration of 50 nM siRNA. Cells were incubated with the treatment for 24 and 48 hrs, and the cells and media were collected for subsequent evaluation.

### RT-PCR analysis

After 24 and 48 hrs of treatment, cells were collected in TriReagent (Sigma-Aldrich) and processed for total RNA extraction according to the classical method. The isolated RNA was analysed for quality (2100 Bioanalyzer, Agilent Technologies, Santa Clara, CA, USA) and quantity (NanoDrop ND-1000 Thermo Scientific, Wilmington, DE, USA) before further processing. All analysed samples had RNAs with a RIN between 9 and 10. 500 ng total RNA were used for cDNA synthesis using FirstStrandcDNA synthesis kit (Hoffmann-La Roche, Basel, Switzerland) following the random hexamer primer protocol. The cDNAs were diluted 1:10 and amplified with TaqMan Master kit (Hoffmann-La Roche) in a LightCycler 480 II thermocycler (Hoffmann-La Roche). Expression levels were quantified by ΔΔCt method using 18S as housekeeping gene.

### ELISA

The levels of PDGFBB protein were determined by standard sandwich ELISA. Briefly, clear micro high affinity ELISA plates (DY990; R&D Systems, Minneapolis, MN, USA) were coated overnight at 4°C with 100 μl of 4 μg/ml PDGFBB capture antibody (MAB1739; R&D Systems). The plates were blocked with a 1% BSA solution for 1 hr and incubated with 100 μl of cell media or serial dilutions of PDGFBB protein (P3201-10UG; Sigma-Aldrich) for 2 hrs followed by 1 hr incubation with 100 μl of 0.5 μg/ml PDGFBB detection antibody (BAF 220; R&D Systems) to allow antigen–antibody reaction. Between each step, the plates were washed three times with 1× Washing Buffer (WA126; R&D Systems). The plates were incubated with Streptavidine-HRP conjugate (DY 998; R&D Systems) at a concentration of 1:200 for 30 min. and with the Substrate (DY 999; R&D Systems) for another 20 min. The reaction was stopped by the addition of 50 μl of H_2_SO_4_ 2N. Optical densities were read with Biotek Synergy 2 spectrophotometer at 450 nm.

### Microarray expression profiling and analysis

Total RNA isolated as described above was purified with RNeasy Mini Kit (Qiagen, Hilden, Germany) and used for gene expression profiling using 4 × 44k Whole Human Genome Oligo Microarray (Agilent Technologies, Santa Clara, CA, USA) with two-colour design. As we have targeted only one gene, we did not expect to observe major differences in gene expression, and two-colour microarray analysis has been observed to be more sensitive to small fold changes. A quantity of 100 ng total RNA from negative control- and PDGFBB siRNA-treated cells obtained in three independent experiments were used for microarray probe synthesis. Technical replicates of each sample were used for hybridization control (dye-swap), each sample was labelled with Cy3 and Cy5 in independent experiments using *Low Input Quick Amp Labeling Kit* (Agilent Technologies), according to the manufacturer's instructions. The slides were scanned with Agilent Technologies scanner G2505B US45102867 and image processing was performed with Feature Extraction software v. 10.5.3 (Agilent Technologies).

Microarray data analysis was performed in R (www.bioconductor.org). Background and foreground intensity ratios were computed taking log2 ratios of intensities for red (R) and green (G) fluorescence channels (M values). Intra-slide normalization was carried out using Loess regression. Data were further subjected to inter-slide normalization by quantile method. Median M values for duplicate spots were computed and used in class comparison analysis. Differentially expressed genes between PDGFBB siRNA- and negative control-treated cells were selected in R using moderated t-statistics. A gene was considered differentially expressed if M value for that gene was lower than −0.38 or greater than 0.38 (−1.3 ≥ fold regulation ≥ 1.3) and p-value adjusted for multiple testing <0.05 (Benjamini and Hochberg method).

### Cell proliferation

Ca Ski and HeLa cells (2 × 10^4^) were seeded on 96-well plates and treated as described above. After 24 and 48 hrs of incubation, the cells were stained with MTT and incubated 1 hr for dye incorporation. Blue formazans were dissolved in DMSO and quantified with Tecan Sunrise plate reader.

### Apoptosis evaluation

The cells treated as described above were trypsinized, collected, stained with Anexinn V-biotin Apoptosis Detection kit (Calbiochem, Merck Millipore, Darmstadt, Germany) and quantified by on-chip flow cytometry. The number of apoptotic cells was assessed with Agilent Lab-on-a-chip Bioanalyzer 2100, as percent of apoptotic cells in live cells.

### Migration assay

The effect of PDGFBB inhibition on the migration property of cervical cancer cells was determined using the BD Falcon 3 μm FluoroBlok 96-Multiwell Insert Systems transwell migration assay in co-culturing conditions. HeLa and Ca Ski cells were fluorescently labelled using PKH26 Red Fluorescent Cell Linker Kits (Sigma-Aldrich). This staining ensures maintenance of fluorescence of live cells for a longer period of time. Cells were trypsinized, 1 × 10^6^cells were washed twice with PBS, centrifuged (110 g, 5 min.) and the cell pellet was resuspended in 1 ml Diluent C and 1 ml of Dye Solution (4 μl of PKH26/ml). The staining was stopped after 5 min. by adding 10 ml of complete medium containing 10% foetal calf serum and cells were centrifuged for 10 min. at 1000 r.p.m. Another two washing steps were performed with 10 ml of complete medium. Cells were counted and 1.25 × 10^4^ Ca Ski and HeLa cells were resuspended in Opti-Mem, plated on the top chamber of the cell culture inserts and treated with siRNA as described above. On the bottom wells was added either 10% serum-containing medium, 10^4^ HUVEC cells in serum-containing or serum-free medium as chemoattractants. After 24 and 48 hrs of incubation, the fluorescence intensity of migrated cells was read in fluorescence at 540–620 nm with Biotek Synergy 2 microplate according to the manufacturer's protocol. The influence of co-culturing on HUVEC cells proliferation was monitored by treating the cells with Fluorescein Diacetate and quantified at 492 nm.

### Invasion assay

Ca Ski and HeLa cells were treated with negative control- and PDGFBB-siRNA for 48 hrs, trypsinized and resuspended in Opti-Mem medium. 10^5^ of treated cells were plated in the top chamber of the cell culture inserts (6.5 mm diameter insert, 8.0 μm pore size, Corning Life Sciences, Amsterdam, The Netherlands) pre-treated with 1:10 diluted Matrigel (Sigma-Aldrich). Ten percentage of serum-containing medium was added in the bottom chamber to stimulate cell invasion. After incubation for 24 and 48 hrs, the cell inserts were removed from the plate and cells that did not migrate were mechanically removed with a cotton swab. Invaded cells were fixed in ice-cold methanol and the membranes were cut and mounted on microscope slide with DAPI mounting medium. The migrated cells were examined using inverted phase fluorescence Zeiss Axiovert microscope (Carl Zeiss Microscopy GmbH, Jena, Germany) and counted from five randomly selected fields in a blind way.

### Adhesion to endothelium

Confluent monolayers of HUVEC cells were cultured for 24 hrs on plastic cover slips pre-treated with 1% gelatin for 30 min. After 48 hrs of siRNA treatment, Ca Ski and HeLa cells were harvested, counted and added to the coverslips. The cells were allowed to adhere to the HUVEC monolayer for 50 min. at 37°C, after which the non-adherent HeLa cells were thoroughly washed with PBS–Alb, fixed in 4% paraformaldehyde for 15 min. at RT and stained with Giemsa solution for 5 min. The cover slips were washed with PBS, allowed to air-dry and mounted on microscope slides. Using light microscopy, the number of cells adherent to the monolayer was counted in five fields of view per well selected at random.

### Capillary tube formation

To study the influence of PDGFBB inhibition in cervical cancer cells on endothelial tube formation, we used a protocol adapted from Gulec and Woltering 12 by cultivating HUVEC cells in fibrin gels. Serum-free endothelial growth medium consisted of Medium 199/CS-C medium(1/3), Penicillin 100 UI/ml, Streptomycin (100 μg/ml), amphotericin B 2.5 μg/ml, 1% endothelial cell growth factor (×100) with a final concentration of 3 mg/ml fibrinogen and 0.05 U/ml thrombin (all reagents from Sigma-Aldrich) was added in 4-well chamber slides (300 μl/well) and allowed to clot for 30 min. in a humidified incubator at 37°C. 10^4^ HUVEC cells were suspended in 800 μl conditioned media collected from 24 and 48 hrs siRNA- treated Ca Ski and HeLa cells. These cells were added on the top of fibrin substrate in each well and examined using an inverted phase Zeiss Axiovert microscope. Image acquisition was performed with an AxioCam MRC camera.

### Statistical analysis

Each experiment was performed at least three times. All the data are presented as mean ±SEM. Differences were assessed by *t*-test using the Prism statistical program. *P* *<* 0.05 was considered to be statistically significant (*P < 0.05, **P < 0.01, ***P < 0.001).

## Results

### PDGFBB down-regulation induces changes in the gene expression profile of cervical cancer cells

**Fig 1:**
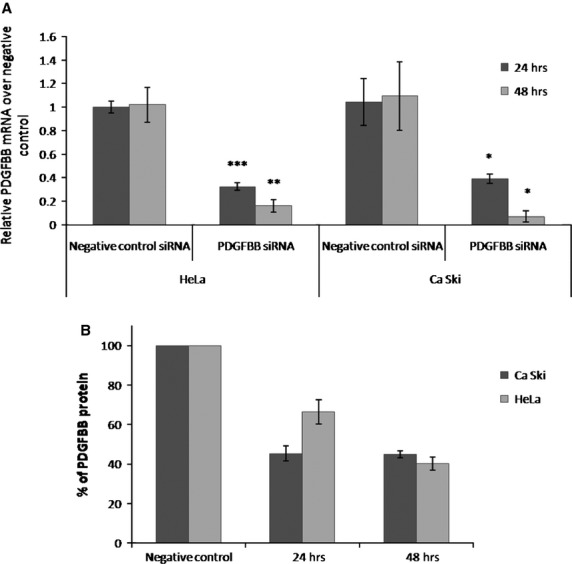
Quantification of siRNA treatment effects on PDGFBB expression levels. Ca Ski and HeLa cells were treated with negative control- and PDGFBB-siRNA for 24 and 48 hrs and monitored for mRNA and protein expression. (A) Relative mRNA PDGFBB levels upon siRNA treatment; (B) % of protein levels remained upon siRNA treatment.

Ca Ski and HeLa cells were treated with negative control- and PDGFBB-siRNA for 24 and 48 hrs. RT-PCR analysis of PDGFBB mRNA upon treatment confirmed PDGFBB knock-down by 70% in the first 24 hrs of treatment and over 85% after 48 hrs (Fig.1A). ELISA quantification of PDGFBB protein levels upon treatment confirms protein knock-down to 60% at 48 hrs after treatment (Fig.1B).

To investigate the early molecular changes induced by PDGFBB inhibition in cervical cancer, we performed class comparison analysis of the 24 hrs PDGFBB siRNA-treated *versus* negative control-treated Ca Ski cells. Squamous carcinoma histology is the most common type of cervical cancer; therefore, we chose this cell line for in-depth transcriptional studies. While qRT-PCR analysis showed ≈3-fold mRNA down-regulation of PDGFBB expression upon PDGFBB siRNA treatment, in microarray analysis we identified a corresponding 1.3-fold regulation of PDGFBB expression. Differences between microarray and qRT-PCR expression levels have been previously documented 13,14; however, as qRT-PCR is considered to be the standard method, we considered of interest genes with fold regulation above 1.3 threshold. Using an M value cut-off of 0.38 (1.3-fold regulation) and an adjusted *P* < 0.05, we found 671 differentially expressed genes in the microarray experiment.

To assess the molecular and cellular functions 15 of the identified genes, we performed the Ingenuity Pathway Core Analysis. Of the 671 gene identified, 579 were mapped in Ingenuity Knowledge Base, and the unmapped genes were discarded from further analysis. Of the total number of differentially expressed genes modulated by PDGFBB knock-down, 292 were down-regulated and 287 were up-regulated. The top up and down-regulated genes (Table1) as a result of PDGFBB inhibition on Ca Ski cells, were found to code for enzymes, transcription regulators, kinases and growth factors, all with different cellular localizations.

**Table 1:** Top up- and down-regulated genes induced by PDGFBB knock-down in Ca Ski cells

Symbol	Fold change	Gene name	Type(s)
TOP1MT	2.626	Topoisomerase (DNA) I, mitochondrial	Enzyme
HIPK3	2.389	Homeodomain interacting protein kinase 3	Kinase
HOMER1	2.33	Homer homolog 1 (Drosophila)	Other
LSM14A	2.303	LSM14A, SCD6 homolog A (S. cerevisiae)	Other
EMP2	2.296	Epithelial membrane protein 2	Other
YWHAH	2.205	Tyrosine 3-monooxygenase/tryptophan 5-monooxygenase activation protein, eta polypeptide	Transcription regulator
GOLGA4	2.095	Golgin A4	Other
POLR3E	2.085	Polymerase (RNA) III (DNA directed) polypeptide E (80kD)	Transcription regulator
THG1L	2.009	tRNA-histidineguanylyltransferase 1-like (S. cerevisiae)	Enzyme
ILF3	1.956	Interleukin enhancer binding factor 3, 90 kD	Transcription regulator
DKK1	−3.237	Dickkopf 1 homolog (Xenopuslaevis)	Growth factor
DEFB4A/DEFB4B	−2.789	Defensin, beta 4A	Other
NEURL3	−2.614	Neutralized homolog 3 (Drosophila) pseudogene	Other
ANKRD22	−2.279	Ankyrin repeat domain 22	Transcription regulator
GLS	−2.251	Glutaminase	Enzyme
NAP1L1	−2.182	Nucleosome assembly protein 1-like 1	Other
DAAM1	−2.059	Dishevelled associated activator of morphogenesis 1	Other
GLS	−1.991	Glutaminase	Enzyme
DNAJA1	−1.989	DnaJ (Hp40) homolog, subfamily A, member 1	Other
MYCBP	−1.971	c-myc binding protein	Transcription regulator
PGK1	−1.934	Phosphoglycerate kinase 1	Kinase

The main functions regulated by the identified genes are related to cell death and survival, cell growth and proliferation, cardiovascular system development, cell-to-cell signalling and cellular movement (Table2). Several genes were assigned to signal cancer and ECs proliferation, death and survival, adhesion and cell motility, development of epithelial and endothelial tissues, development of blood vessels, epithelial-mesenchymal transition, all functions previously associated with PDGFBB signalling.

**Table 2:** Selected functions modulated by the identified genes

Function category (no of molecules)	*P*-value	Functions of interest	Selected genes for further validation
Cell death and survival (178 molecules)	8.83E^−11^–7.5E^−3^	Cell death, apoptosis, cell survival	NRAS, CYR61, EDN1, IL15, NRG1, LGALS3, PLAUR, LRIG1, CSF2, DKK1, DNAJA1, HIPK3, EMP2, THG1L, ILF3
Tissue development (104 molecules)	2.4E^−9^–6.8E^−3^	Development of epithelial, endothelial tissues, endothelial cell development and proliferation, adhesion of epithelial cells, tubulation of epithelial tissue, detachment of cells	NRAS, CYR61, EDN1, IL15, NRG1, LGALS3, PLAUR, CSF2, DKK1
Cellular growth and proliferation (174 molecules)	2.75E^−8^–7.56E^−3^	Tumour and endothelial cell proliferation	NRAS, CYR61, EDN1, IL15, NRG1, LGALS3, PLAUR, LRIG1, CSF2, DKK1, DNAJA1, MYCBP, PGK1, EMP2, THG1L, ILF3
Cardiovascular system development and function (84 molecules)	3.71E^−8^–6.76E^−3^	Endothelial cell development and proliferation, development of blood vessels, cell movement of endothelial cells	NRAS, CYR61, EDN1, IL15, NRG1, LGALS3, PLAUR, CSF2, DKK1, PGK1
Cellular development (150 molecules)	5.8E^−7^–7.56E^−3^	Development of epithelial, endothelial cells	NRAS, CYR61, EDN1, IL15, NRG1, LGALS3, PLAUR, LRIG1, CSF2, DKK1, MYCBP, PGK1
Organismal development (121 molecules)	5.8E^−7^–7.97E^−3^	Development of endothelial cells and blood vessels	NRAS, CYR61, EDN1, IL15, NRG1, LGALS3, PLAUR, LRIG1, CSF2, DKK1,DNAJA1, PGK1,ILF3
Cell-to-cell signalling and interaction (61 molecules)	1.01E^−6^–7.84E^−3^	Adhesion of epithelial and endothelial cells	NRAS, CYR61, EDN1, IL15, LGALS3, PLAUR, CSF2, DKK1
Cell morphology (119 molecules)	1.82E^−6^–6.76E^−3^	Tubulation of cells	NRAS, CYR61, EDN1, IL15, NRG1, LGALS3, PLAUR, CSF2, DNAJA1
Cellular movement (96 molecules)	3.8E^−5^–7.99E^−3^	Tumour cell movement, invasion of cells, migration of tumour and endothelial cells	NRAS, CYR61, EDN1, IL15, NRG1, LGALS3, PLAUR, LRIG1, CSF2, DKK1, DNAJA1

To assess the reliability of microarray results, we considered several genes (Table3) as candidates for validation by qRT-PCR as following: highly, intermediately and low-expressed genes. Chosen genes were selected from top and down-regulated genes as well as randomly, according to their known functions as modulators for proliferation, death, survival, motility and adhesion. Our results show that most of the genes were validated by qRT-PCR analysis, the ones that did not present statistically significant differences, could be among the 5% false rate discovery genes (*P* < 0.05). We further tested the consistency of these results between the two histological types of cervical cancer, by analyzing the expression of the same genes in the second cervical cancer cell line (HeLa cells). To be able to compare the results between the two cells lines, we had to consider similar levels of protein knock-down; therefore, gene expression analysis in HeLa cells was performed at 48 hrs time-point (Fig.1). With the exception of PLAUR and CSF2 genes which were up-regulated in Ca Ski cells and down-regulated in HeLa cells upon PDGFBB inhibition, the genes showed the same pattern of expression between the two cell lines. However, some genes that were not significant in Ca Ski cells were found to be significant in HeLa cells, while some genes that were significant in Ca Ski cells were not significant in Hela cells (Table3).

**Table 3:** Microarray data validation by qRT-PCR

Gene	Localization	Fold regulation		
		Microarray	Ca Ski 24 hrs	HeLa 48 hrs
HIPK3	Nucleus	2.389	2.28^*^^*^^*^	2.44^ns^
EMP2	Plasma membrane	2.296	1.85^*^	3.10^ns^
THG1L	Cytoplasm	2.009	2.23^*^	3.57^ns^
ILF3	Nucleus	1.956	1.38^*^^*^	2.19^ns^
PLAUR	Plasma membrane	1.421	1.64^ns^	−1.47^*^
CSF2	Extracellular space	1.416	1.97^*^^*^	−1.98^ns^
LRIG1	Extracellular space	1.375	1.21^ns^	1.50^*^
LGALS3	Extracellular space	1.302	1.37^ns^	1.89^*^^*^
DKK1	Extracellular space	−3.237	−5.38^*^	−2.44^ns^
DNAJA1	Nucleus	−1.989	−1.70^*^	−1.38^ns^
MYCBP	Nucleus	−1.971	−2.40^*^	−2.98^ns^
PGK1	Cytoplasm	−1.934	−2.03^*^	−3.71^*^
NRAS	Plasma membrane	−1.812	−1.66^*^	−2.83^ns^
CYR61	Extracellular space	−1.711	−1.99^*^	−2.98^*^
EDN1	Extracellular space	−1.546	−1.96^ns^	−1.56^*^
PDGFBB	Extracellular space	−1.305	−2.91^*^	−6.70^*^^*^

### PDGFBB inhibition does not influence cervical cancer cells proliferation, death or motility

**Fig 2:**
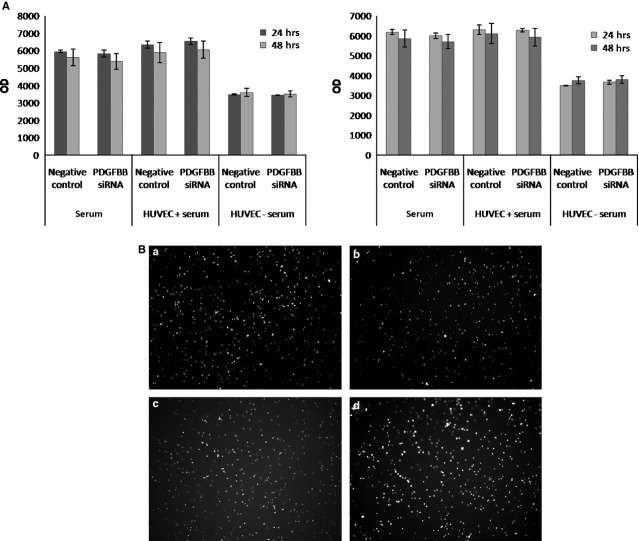
Influence of PDGFBB inhibition on cervical cancer cells (A) migration: Ca Ski (left) and HeLa (right) cells were fluorescently labelled, plated in the top chamber of cell culture inserts, treated with negative control- and PDGFBB-siRNA and allowed to migrate towards different stimulators for 24 and 48 hrs, (B) invasion: Ca Ski and HeLa cells were treated with negative control- and PDGFBB-siRNA for 48 hrs on 12 well plates, harvested and plated on the top chamber of Matrigel pre-treated cell culture inserts and allowed to invade for 24 hrs. (a and b) negative control- and PDGFBB siRNA-treated Ca Ski cells; (c and d) negative control- and PDGFBB siRNA-treated HeLa cells microscopic visualization of invaded cells.

According to IPA analysis, the genes identified as differentially expressed upon PDGFBB inhibition are associated to several cellular functions; therefore, we investigated if these changes in gene expression modulate any biological activity in cervical cancer cells. Proliferation and apoptosis studies showed no significant changes in either viable or apoptotic cell number in Ca Ski or HeLa cell lines (data not shown) upon PDGFBB siRNA treatment. Furthermore, because cervical cancer cells are known to have increased metastatic potential, we investigated if siRNA treatment affected cells motility by performing two *in vitro* assays under different conditions to simulate the metastatic process. As shown in Figure2, PDGFBB knock-down did not affect cells ability to migrate towards different chemoattractants (serum media, ECs) or invade through Matrigel extracellular matrix.

### PDGFBB knock-down induces changes in cervical cancer cell adhesion to endothelium

**Fig 3:**
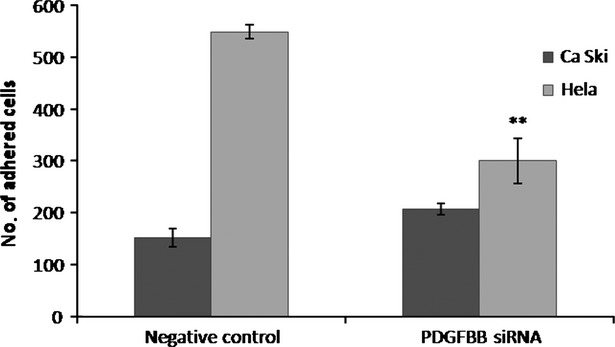
Adhesion of cervical cancer cells to an endothelial cells monolayer. Ca Ski and HeLa cells were treated with negative control- and PDGFBB-siRNA for 48 hrs on 12 well plates, harvested and seeded on top of a HUVEC cells monolayer and allowed to adhere for 50 min. The adhered cells were counted in five different fields

During the metastatic process, tumour cells reach additional sites *via* bloodstream where they establish secondary tumours. To extravagate into the target tissues, tumour cells have to establish tight adhesive connections to endothelium to survive the blood flow. Our results show that by inhibiting PDGFBB, we modulated cervical cancer cells ability to adhere to an ECs monolayer. The observed effects were cell line specific: in Ca Ski cells, PDGFBB inhibition lead to an approximately 40% increase in the number of cells adhered, whereas in HeLa cells, the number of adhered cells decreased by 45% (Fig.3).

### PDGFBB down-regulation in cervical cancer cells leads to paracrine inhibition of EC proliferation and aggregation

**Fig 4:**
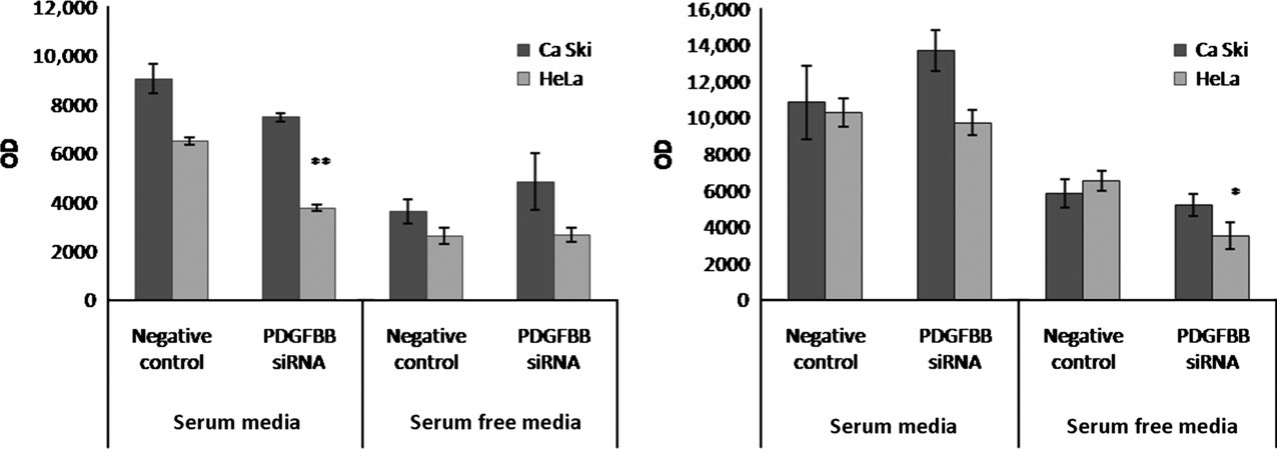
Paracrine effects of siRNA-treated cervical cancer cells on HUVEC cells proliferation in co-culturing conditions. Ca Ski and HeLa cells were seeded on the top chamber of cell culture inserts and treated with negative control- and PDGFBB-siRNA. Fluorescein Diacetate labelled HUVEC cells were added to the bottom chamber, and cells proliferation was monitored for 24 (left) and 48 (right) hrs.

To further delineate the biological functions induced by PDGFBB knock-down in cervical cancer cells, we tested the paracrine effects on ECs. To test this, we established a co-culture system of cervical cancer cells with HUVEC cells. We investigated the effects of co-culturing on ECs proliferation in the presence and absence of serum. Our results show that after 24 hrs, PDGFBB siRNA treated Ca Ski and HeLa cells reduced the proliferation rate of ECs grown in serum-containing medium (Fig.4), although the effect was not significant for Ca Ski co-cultures (*P* < 0.06). This effect was lost after 48 hrs, however, at this time, we observed a 45% decrease in proliferation of ECs grown in serum-free media in the presence of HeLa cells.

**Fig 5:**
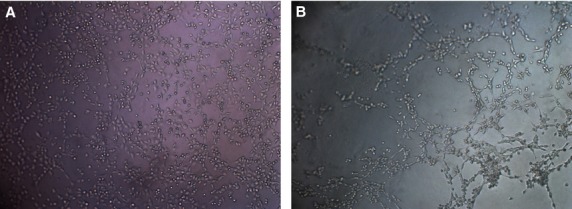
Control of tube formation ability of HUVEC cells grown on fibrin gels. Cells were seeded in pro-angiogenic serum-free medium in 4-well slides pre-coated with fibrin gels. Within 5 hrs HUVEC cells started to assembly into a netlike array of capillary tubes (A), and by 24 hrs capillary tubes were fully developed (B).

**Fig 6:**
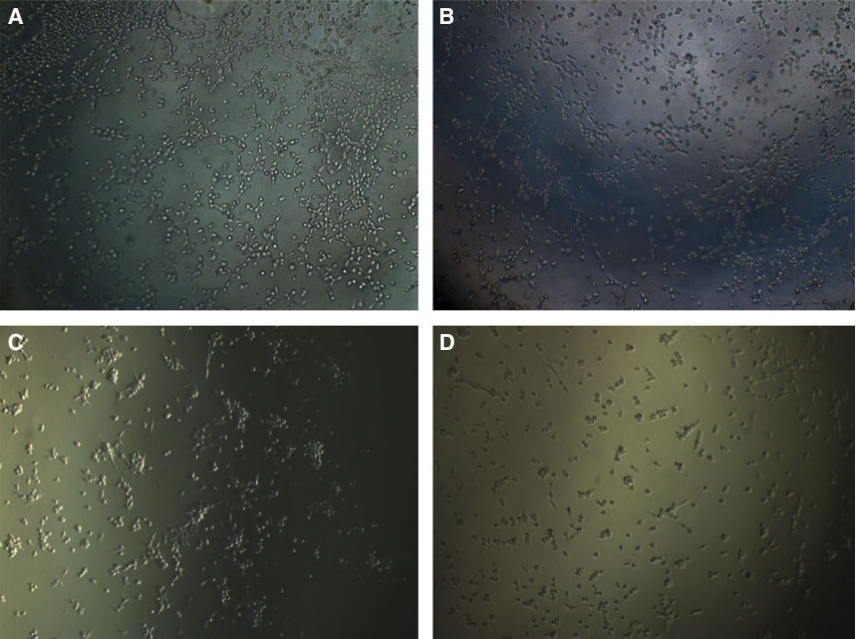
Influence of Ca Ski-treated cells conditioned media on HUVEC cells ability to form capillary tubes. Ca Ski cells were treated for 24 hrs with siRNA, the media were collected and used to culture HUVEC cells in 4-well slides pre-coated with fibrin gels. After 5 hrs, the HUVEC cells grown in negative control siRNA conditioned media apparently started to form capillary like structures (A) while the cells grown in PDGFBB siRNA conditioned media showed reduced ability to develop capillary tubes (B). At 24 hrs, the cells grown in negative control siRNA conditioned media appeared as cellular aggregates (C) instead of capillary tubes, while the cells grown in PDGFBB siRNA conditioned media remained as singular cells (D).

**Fig 7:**
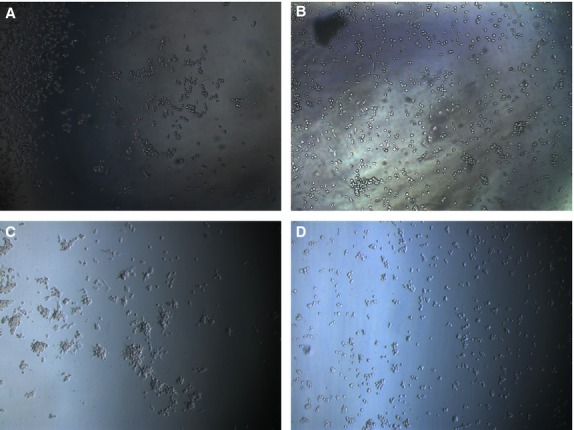
Influence of HeLa-treated cells conditioned media on HUVEC cells ability to form capillary tubes. HeLa cells were treated for 48 hrs with siRNA, the media were collected and used to culture HUVEC cells in 4-well slides pre-coated with fibrin gels. When cultured in negative control siRNA conditioned media, the HUVEC cells showed no ability to form capillary tubes neither after 5 hrs (A) or 24 hrs (C) of culturing. However, the cells show clear aggregation tendencies, which seems to be abolished when the cells were cultured in PDGFBB siRNA conditioned media (B and D).

The paracrine effects of PDGFBB inhibition on tube formation ability were investigated by culturing HUVEC cells on fibrin gels. First, we tested the ability of HUVEC cells to form capillary tubes in pro-angiogenic media (Fig.5). After 5 hrs of culture, the cells started to assemble into capillary tube-like structures (Fig.5A), while at 24 hrs the tubes were fully formed (Fig.5B). Ca Ski and HeLa cells were treated for 24 and 48 hrs with siRNA, the conditioned media were collected, and further used to culture HUVEC cells. We observed that, unlike the HUVEC grown in angiogenic media, in Ca Ski and HeLa conditioned media the cells rather assemble into cellular aggregates than capillary tubes. We believe that this is due to the use of Opti-Mem media, which contains insufficient pro-angiogenic factors, which are necessary to activate the HUVEC cells to form capillary tubes. Five hours after cell seeding in Ca Ski cells conditioned media, we observed a tendency of HUVEC cells to assembly into capillary tubes (Fig.6A); however, after 24 hrs the cells appeared as cellular aggregates rather than tube-like structures (Fig.6C). However, the cells grown in conditioned media collected from PDGFBB siRNA treated Ca Ski cells show inhibited tube formation ability at 5 hrs (Fig.6B) and appear as single cells after 24 hrs (Fig.6D). The ECs grown in the media collected from negative control siRNA-treated HeLa cells showed low tube formation ability both at 5 (Fig.7A) and 24 hrs (Fig.7C), the cells being assembled into cellular aggregates. However, the cells grown in media collected from PDGFBB siRNA treated HeLa cells (Fig.7B and D) appeared to have reduced tendencies to aggregate.

## Discussions

Despite the clinical benefits of targeting the PDGF signalling as anticancer therapy, the mechanisms responsible for this success are yet not well understood. PDGFBB has been shown to mainly act on PDGFR-expressing vascular mural cells and stromal cells, and usually does not affect cells that do not express detectable levels of PDGFRs, such as ECs or malignant cells. However, PDGFBB induce angiogenesis without recruiting ECs, implying that the angiogenic effect of PDGFBB is mediated through complex regulatory mechanisms involving intimate interplay between several signalling pathways. Moreover, PDGF signalling is often activated as a compensatory mechanism to alternative angiogenic pathways because of acquired resistance to current antiangiogenic therapy. PDGF signalling may be activated upon anti-VEGF therapy 16; however, dual targeting of VEGF and PDGF signalling showed only little improvement of anti-tumour activity 17. Thus, the role of PDGFBB in tumour angiogenesis remains elusive and controversial 18. In this study, we investigated the anti-tumour and anti-angiogenic potential of PDGFBB in cervical cancer by studying the signalling mechanism of PDGFBB in two HPV-positive histological types of cervical cancer cells.

We provide evidence that at molecular level, the impairment of PDGFBB signalling by gene knock-down leads to rapid alterations of the transcriptome with significant changes in gene expression in squamous cervical carcinoma Ca Ski cells. Of the differentially expressed genes, only few have been previously associated or investigated in cervical cancer. For example, THG1L which has been up-regulated by PDGFBB inhibition has been shown to modulate cell cycle progression and cell proliferation of cervical cancer cells 19. Down-regulation of PGK in cervical cancer cells affects triphosphate metabolites formation, which incorporates into cellular or viral DNA, leading to chain termination or inhibition of viral reverse transcriptase or DNA polymerase 20. Knock-down of ILF3 expression in cervical cancer cells increased viral polymerase complex activity and virus replication suggesting that ILF3 negatively affects viral replication by down-regulating both viral genome replication and mRNA transcription in infected cells 21. In our study, ILF3 has been up-regulated, suggesting that PDGFBB indirectly controls viral replication in cervical cancer cells. High levels of DKK1 protein have been previously associated with cervical cancer 22; therefore, DKK1 down-regulation by PDGFBB inhibition might have anti-tumour effects, however, the potential biological role of this molecule has not yet been addressed.

Functional analysis of the microarray data showed that the genes altered by PDGFBB knock-down are involved in maintaining cells proliferation, death, survival, motility and adhesion. Further analysis of the biological effects of PDGFBB signalling impairment might have on cervical cancer cells did not show any significant differences in growth, apoptosis, migrations or invasion. We hypothesize that PDGFBB signalling is compensated through a fine-tuning of molecules with antagonistic effects. Therefore, the lack of cellular responses seems to be maintained by shifting between signalling pathways. Based on previously described biological effects and expression levels in our study, NRAS 23 and EMP2 24 are predicted to increase cervical cancer cells apoptosis, while HIPK3 25, DKK1 26, THG1L 19 should decrease apoptosis. Moreover, NRAS 27, CSF2 28 and MYCBP 29 should promote cell growth while DKK1 30 and THG1L 19 levels of expression might lead to proliferation inhibition. The antagonistic effects of these genes could explain how cells may adapt to impaired PDGFBB signalling by turning on and off signalling molecules to prevent and balance apoptosis and cellular growth.

Cell motility is a primordial step in tumour invasion and increased adherence and migration is the first process that indicates the metastatic potential of a tumour cell. Although PDGBB knock-down leads to DKK1 and NRAS inhibition which have been showed to induce migration 31 and invasion 22 in some types of cells, we did not see any change in cells ability to migrate or invade through Matrigel. However, we observed that PDGFBB knock-down lead to significant changes in cervical cancer cells adherence to EC monolayer. Re-establishment of adhesive connections to endothelium is required for tumour cells to be able to extravasate from the bloodstream and form new tumours 32. PDGFBB seems to have pleiotropic effects, PDGFBB siRNA treatment led to increased Ca Ski cells adherence, but reduced the adherence of HeLa cells to ECs (Fig.3). Adherence of tumour cells to endothelium is mainly mediated through integrins, which have been shown to bind CYR61 33 and activate adhesion and signalling receptors. However, as CYR61 was under-expressed in both cell lines and the adhesion effects are opposite; other molecules should be responsible for the different behaviour between Ca Ski and HeLa cells. Ca Ski cells are derived from an epidermoid carcinoma of the cervix metastatic to the small bowel and while adenocarcinoma HeLa cells also present aggressive behaviour, they could present different adhesion mechanisms, which could explain the observation that some of the analysed genes were not significantly expressed in HeLa cells. From the validated genes, there were some genes that had inverse expression levels or were significantly expressed only in one of the cell line (PLAUR, CSF2, LGALS3) and although their expression has been previously associated with cell–cell adhesion, 34,35 additional investigations are necessary to see if any of these genes are responsible for the different adhesive effects between the two cell lines. This behaviour could also be directly dependent on cells morphology, HeLa cells in standard conditions of cultivation on plastic dishes display an epithelial-like morphology and adhere tightly to ECM, while Ca Ski cells are more round and less adherent. Also, HPV viremia could be a factor in these cells adhesion mechanism, as Ca Ski cells have been shown to have 10 times more HPV DNA copies/cell than HeLa cells 36 and HPV has been showed to modulate cells adherence 37 and PDGF expression 38.

When tested for paracrine effects on ECs, PDGFBB siRNA-treated cervical cancer cells decreased ECs proliferation in HeLa co-cultures and conditioned media from both cell lines reduced cellular aggregation in the tube formation assay. Although, previous reports have shown that ECs do not express PDGF receptors and that PDGFBB signalling cascade is only activated upon receptor binding, we conclude that the effect we see is mediated through extracellular molecules that are released by cervical cancer cells upon PDGFBB inhibition. Molecules such as CSF2, LRIG1, LGALS3, DKK1, CYR61, EDN1 and IL15 have been shown to be exported outside the cell and trigger paracrine signalling in surrounding cells. For example, EDN1 increases in a dose-dependent manner proliferation of cultured HUVEC cells 39 and tube formation of lymphatic ECs 40, while CSF2 has been shown to increase proliferation of endothelial progenitor cells 41. A study similar to ours showed that transcriptional suppression of PLAUR in glioma cells leads to reduced tumour-induced migration and proliferation of ECs 42. CYR61, which is significantly down-regulated in both cell lines upon PDGFBB inhibition, has been shown to increase tube formation of cultured unactivated HUVEC cells 43 a process that is mediated by human Integrin complexes 44. Moreover, in fibrin gel, human CYR61 protein increased sprouting of ECs 45. PGK1 protein has been shown to decrease angiogenesis of blood vessels and reduces tumour formation 46 and DKK1 protein increases disruption of blood vessels and reduces proliferation and migration of microvascular ECs 47. DKK1-expressing tumours are bigger with higher vascular density and little attachment of VSMCs/PCs to ECs. DKK1 expression leads to β-catenin down-regulation in ECs, which has been shown to induce PDGFBB expression 48. In our study, a similar but inverse mechanism of PDGFBB dependent regulation of DKK1 could explain down-regulated levels of DKK1 upon PDGFBB inhibition and reduced ECs proliferation and tube formation ability. In kidney injury, however, Ren *et al*. 49 have recently showed that DKK1 inhibits pericyte activation, detachment, and transition to myofibroblasts, predominantly by inhibiting PDGF.

Overall, our findings bring further insight into the PDGFBB complex regulation of tumourigenic and angiogenic potential of cervical cancer cells. PDGFBB knock-down resulted in a rapid transcriptional activity that maintains cervical cancer cell proliferation, survival and motility. The autocrine effects of PDGFBB signalling seem to be limited to cells ability to adhere to ECs, and they are dependent on the cell line. The lack of PDGFRs expression on ECs suggests that PDGFBB signalling impairment in cervical cancer cells reduces angiogenesis through an indirect paracrine signalling mechanisms. In conclusion, we believe that the understanding of the molecular mechanisms of interplays between PDGFBB cascade and other signalling pathways is crucial for the development of multi-targeting therapeutic agents to achieve synergistic anti-tumour and anti-angiogenic effects.
